# The origin and underlying driving forces of the SARS-CoV-2 outbreak

**DOI:** 10.1186/s12929-020-00665-8

**Published:** 2020-06-07

**Authors:** Shu-Miaw Chaw, Jui-Hung Tai, Shi-Lun Chen, Chia-Hung Hsieh, Sui-Yuan Chang, Shiou-Hwei Yeh, Wei-Shiung Yang, Pei-Jer Chen, Hurng-Yi Wang

**Affiliations:** 1 Biodiversity Research Center, Academia Sinica, Taipei, Taiwan; 2 Graduate Institute of Clinical Medicine, College of Medicine, National Taiwan University, Taipei, Taiwan; 3 Department of Life Science, National Taiwan Normal University, Taipei, Taiwan; 4 Department of Forestry and Nature Conservation, Chinese Culture University, Taipei, Taiwan; 5 Department of Clinical Laboratory Sciences and Medical Biotechnology, College of Medicine, National Taiwan University, Taipei, Taiwan; 6 Department of Microbiology, College of Medicine, National Taiwan University, Taipei, Taiwan; 7 Institute of Ecology and Evolutionary Biology, National Taiwan University, Taipei, Taiwan

**Keywords:** Positive selection, Population genetics, Coronavirus, Mutational bias

## Abstract

**Background:**

SARS-CoV-2 began spreading in December 2019 and has since become a pandemic that has impacted many aspects of human society. Several issues concerning the origin, time of introduction to humans, evolutionary patterns, and underlying force driving the SARS-CoV-2 outbreak remain unclear.

**Method:**

Genetic variation in 137 SARS-CoV-2 genomes and related coronaviruses as of 2/23/2020 was analyzed.

**Result:**

After correcting for mutational bias, the excess of low frequency mutations on both synonymous and nonsynonymous sites was revealed which is consistent with the recent outbreak of the virus. In contrast to adaptive evolution previously reported for SARS-CoV during its brief epidemic in 2003, our analysis of SARS-CoV-2 genomes shows signs of relaxation. The sequence similarity in the spike receptor binding domain between SARS-CoV-2 and a sequence from pangolin is probably due to an ancient intergenomic introgression that occurred approximately 40 years ago. The current outbreak of SARS-CoV-2 was estimated to have originated on 12/11/2019 (95% HPD 11/13/2019–12/23/2019). The effective population size of the virus showed an approximately 20-fold increase from the onset of the outbreak to the lockdown of Wuhan (1/23/2020) and ceased to increase afterwards, demonstrating the effectiveness of social distancing in preventing its spread. Two mutations, 84S in orf8 protein and 251 V in orf3 protein, occurred coincidentally with human intervention. The former first appeared on 1/5/2020 and plateaued around 1/23/2020. The latter rapidly increased in frequency after 1/23/2020. Thus, the roles of these mutations on infectivity need to be elucidated. Genetic diversity of SARS-CoV-2 collected from China is two times higher than those derived from the rest of the world. A network analysis found that haplotypes collected from Wuhan were interior and had more mutational connections, both of which are consistent with the observation that the SARS-CoV-2 outbreak originated in China.

**Conclusion:**

SARS-CoV-2 might have cryptically circulated within humans for years before being discovered. Data from the early outbreak and hospital archives are needed to trace its evolutionary path and determine the critical steps required for effective spreading.

## Background

A newly emerging coronavirus was detected in patients during an outbreak of respiratory illnesses starting in mid-December of 2019 in Wuhan, the capital of Hubei Province, China [1–3]. Due to the similarity of its symptoms to those induced by the severe acute respiratory syndrome (SARS) and genome organization similarity, the causal virus was named SARS-CoV-2 by the International Committee on Taxonomy of Viruses [4]. As of 3/16/2020, 167,515 cases of SARS-CoV-2 infection have been confirmed in 114 countries, causing 6606 fatalities. As a result, WHO declared the first pandemic caused by a coronavirus on 3/11/2020 (https://www.who.int/emergencies/diseases/novel-coronavirus-2019/situation-reports). As the virus continues to spread, numerous strains have been isolated and sequenced. On 3/18/2020, more than 500 complete or nearly complete genomes have been sequenced and made publicly available.

SARS-CoV-2 is the seventh coronavirus found to infect humans. Among the other six, SARS-CoV and MERS-CoV can cause severe respiratory illness, whereas 229E, HKU1, NL63, and OC43 produce mild symptoms [5]. Current evidence strongly suggests that all human associated coronaviruses originated from other animals, such as bats and rodents [5, 6]. While SARS-CoV-2 shares similar genomic structure with other coronaviruses [7–10], its sequence differs substantially from some of the betacoronaviruses that infect humans, such as SARS-CoV (approximately 76% identity), MERS-CoV (43% identity), and HKU-1 (33% identity), but exhibits 96% similarity to a coronavirus collected in Yunnan Province, China from a bat, *Rhinolophus affinis*. Therefore, SARS-CoV-2 most likely originated from bats [2, 11].

Several issues concerning the origin, time of virus introduction to humans, evolutionary patterns, and the underlying driving force of the SARS-CoV-2 outbreak remain to be clarified [12, 13]. Here, we analyzed genetic variation of SARS-CoV-2 and its related coronaviruses. We discuss how mutational bias influences genetic diversity of the virus and attempt to infer forces that shape SARS-CoV-2 evolution.

## Methods

### Data collection

137 complete SARS-CoV-2 genomes were downloaded from the Global Initiative on Sharing Avian Influenza Data (GISAID, https://www.gisaid.org/) (Supplementary Table 1). Related coronavirus sequences, including those from five related bat sequences (RaTG13, HUK3–1, ZC45, ZXC-21, and GX2013), two pangolins (each from Guangdong (pangolin_2019) and Guangxi (pangolin_2017)), were downloaded from GenBank (https://www.ncbi.nlm.nih.gov/nucleotide/). Nucleotide positions and coding sequences (CDSs) of SARS-CoV-2 were anchored to the reference genome NC_045512. CDS annotations of other coronaviruses were downloaded from GenBank.

### Sequence analyses and phylogeny construction

CDSs were aligned based on translated amino acid sequences using MUSCLE v3.8.31 [14], and back-translated to their corresponding DNA sequences using TRANALIGN software from the EMBOSS package (http://emboss.open-bio.org/) [15]. Nucleotide diversity, including number of segregating sites, Watterson’s estimator of θ [16], and nucleotide diversity (π) [17], was estimated using MEGA-X [18]. MEGA-X was also used for phylogenetic construction. Phylogenetic relationships were constructed using the neighbor-joining method based on Kimura’s two-parameter model implemented in MEGA-X. Number of nonsynonymous changes per nonsynonymous site (dN) and synonymous changes per synonymous site (dS) among genomes were estimated based Li-Wu-Luo’s method [19] implemented in MEGA-X and PAML 4 [20]. The RDP file for the haplotype network analyses was generated using DnaSP 6.0 [21] and input into Network 10 (https://www.fluxus-engineering.com/) to construct the haplotype network using the median joining algorithm. Four haplotype test implemented in DnaSp was applied to test for possible recombination event.

The mutation rate of SARS-CoV-2 and the time to the most recent common ancestor (TMRCA) of virus isolates were estimated by an established Bayesian MCMC approach implemented in BEAST version 1.10.4 [22]. The sampling dates were incorporated into TMRCA estimation. The analysis was performed using the HKY model of nucleotide substitution assuming an uncorrelated lognormal molecular clock [23]. We linked substitution rates for the first and second codon positions and allowed independent rates in the third codon position. We performed two independent runs with 3 × 10^8^ MCMC steps and the results were combined. Log files were checked using Tracer (http://beast.bgio.ed.ac.uk/Tracer). Effective sample sizes were > 300 for all parameters.

## Results

### Molecular evolution of SARS-COV-2 and related coronaviruses

The resulting phylogeny reveals that RaTG13 is the closest relative of SARS-COV-2, followed by pangolin_2019 and pangolin_2017, then CoVZC45 and CoVZXC21, and other SARS-related sequences as outgroups (Supplementary Fig. 1). According to general time reversible model, transition occurred more frequent than transversion with C-T and A-G changes account for 45 and 28%, respectively, of all six types of nucleotide changes. We next estimated the strength of selection for each coding region using the dN and dS. While purifying selection tends to remove amino acid-altering mutations, thus reducing dN and dN/dS, positive selection has the opposite effect, increasing dN and dN/dS [24]. Between SARS-CoV-2 and RaTG13, *orf8* gene exhibits the highest dN (0.032 highlighted in bold in Table 1), followed by *spike* (0.013) and *orf7* (0.011), all above the genome average of 0.007 (Table 1). dS varies greatly among CDSs with the highest of 0.313 in *spike* and the lowest of 0.018 in *envelope* (genome average 0.168). Finally, dN/dS is the highest in *orf8* (0.105), followed by *orf7* (0.061) and *orf3* (0.060), with the genome average of 0.042. Since *spike* shows both high dS and dN, its protein evolution rate (dN/dS) is only 0.040. Thus, while the coronavirus evolved very rapidly, it has actually been under tremendous selective constraint [13].

**Table 1:** Pairwise comparison of nonsynonymous (dN; above slash) and synonymous (dS; below slash) divergence between SARS-CoV-2, RaTG13, and Pangolin_2019 of different coding regions

*Gene*	Length (aa)	SARS-CoV-2 vs RaTG13	SARS-CoV-2 vs Pangolin_2019	RaTG13 vs Pangolin_2019
All	9555	0.007/0.168	0.024/0.469	0.025/0.467
		(0.042)	(0.051)	(0.054)
*orf1a*	4330	0.008/0.166	0.024/0.472	0.023/0.472
		(0.048)	(0.051)	(0.049)
*orf1b*	2692	0.003/0.126	0.008/0.505	0.010/0.515
		(0.024)	(0.016)	(0.019)
*spike*	1219	0.013/0.313	0.068/0.651	0.073/0.680
		(0.040)	(0.104)	(0.107)
RBD of *spike*^*A*^	219	0.055/0.511	**0.023/0.710**	0.058/0.863
		(0.107)	**(0.032)**	(0.068)
*orf3*	274	0.009/0.156	0.019/0.285	0.019/0.261
		(0.060)	(0.066)	(0.072)
*envelope*	75	0/0.018	0/0.037	0/0.018
		(0)	(0)	(0)
*matrix*	221	0.004/0.186	0.010/0.299	0.006/0.317
		(0.021)	(0.033)	(0.019)
*orf6*	60	0/0.099	0.014/0.220	0.014/0.345
		(0)	(0.062)	(0.040)
*orf7*	121	0.011/0.177	0.018/0.275	0.029/0.329
		(0.061)	(0.066)	(0.088)
*orf8*	121	0.032/0.303	0.025/0.362	0.017/0.391
		(0.105)	(0.069)	(0.042)
*nucleocapsid*	415	0.005/0.124	0.011/0.145	0.010/0.125
		(0.042)	(0.076)	(0.080)

Numbers in parentheses are dN/dS ratios throughout this table

*A*: *RBD* Receptor binding domain of *spike*

Spike protein similarity between SARS-CoV-2 and pangolin_2019 led to the idea that the receptor binding domain (RBD) within the SARS-CoV-2 spike protein originated from pangolin_2019 via recombination [25–28]. If that were the case, we would expect the divergence at synonymous sites (dS) to also be reduced in the RBD region. However, while dN in the RBD region is 0.023, approximately one third of the estimate for the rest of the *spike* gene (0.068), dS in the RBD (0.710) is actually slightly higher than in the rest of the *spike* sequence (0.651). This argues against the recombination scenario. We noticed that the dS of the whole *spike* and the RBD, are 2- and 3-fold, respectively, higher than the genome average. Since synonymous sites are typically less influenced by selection, the increased divergence in dS may require further investigation.

### Genetic variation of SARS-CoV-2

**Fig. 1:**
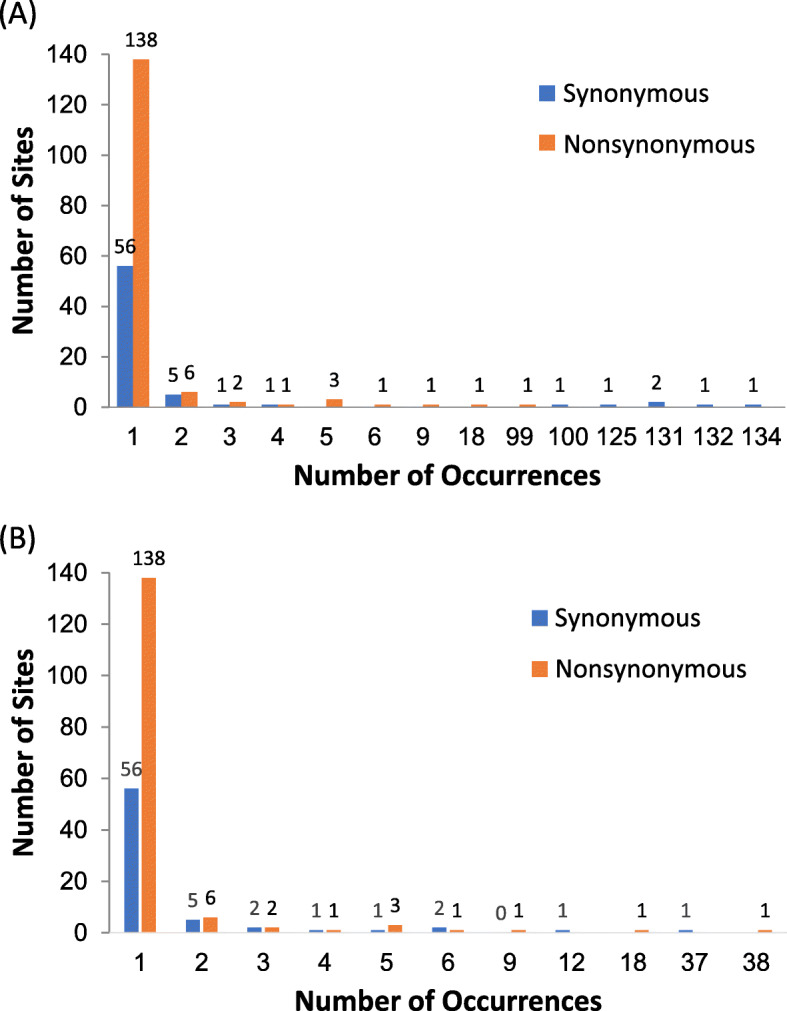
Frequency spectra of SARS-CoV-2. The mutation frequency in 137 SARS-CoV-2 genomes is depicted on the x axis, and the y axis shows the number of sites in which mutations occurred. **a** The derived nucleotides were inferred by referencing SARS-CoV-2 genomes to the RaTG13 genome. **b** The direction of changes was cross-referenced with the haplotype network in Fig. 2

We downloaded 137 SARS-CoV-2 genomes available from GISAID as of 2/23/2019. The coding regions were aligned and 223 mutations were identified with 68 synonymous and 155 nonsynonymous changes. The directionality of changes was inferred based on the RaTG13 sequence. Frequency spectra of both synonymous and nonsynonymous changes are skewed. While the former shows excess of both high and low frequency mutations, the latter mainly exhibits an excess of low frequency changes (Fig. 1a). The excess of low frequency mutations is consistent with the recent origin of SARS-CoV-2 [29]. Both population reduction and positive selection can increase high frequency mutations [30, 31]. However, the first scenario is contradicted by the recent origin of the virus. If positive selection has been operating, we would expect an excess of high frequency non-synonymous as well as synonymous changes. Furthermore, the ratio of nonsynonymous to synonymous (N/S) changes is 2.46 (138/56) among singleton variants, but only 1.23 (16/13) among non-singletons. Both the nonsynonymous frequency spectrum and N/S ratio demonstrate that the majority of amino acid-altering mutations did not reach to high frequency. Thus, evidence for positive selection is limited.

The skew of synonymous variants toward high frequency deserves further discussion, as it relates to the underlying force driving the SARS-CoV-2 outbreak. The puzzle is probably rooted in how high and low frequency mutations are inferred. The results shown in the Fig. 1a are based on an outgroup comparison. The divergence at synonymous sites between SARS-CoV-2 and RaTG13 is 17%, approximately 3-fold greater than between humans and rhesus macaques [32]. With such high level of divergence, the possibility of multiple substitutions cannot be ignored, especially since substitutions in coronavirus genomes are strongly biased toward transitions (see above). Indeed, among all non-singleton mutations listed in Table 2, 62% of the changes are C-T transitions.

**Table 2:** Non-singleton mutations detected across the sampled SARS-CoV-2 genomes

	Genome position	Gene	RaTG13	Pangolin_2017	Pangolin_2019	Major allele	Minor allele	amount of change		
Nonsynonymous								I	II	
A	614	*orf1ab*	G	G	G	G	A	2		H116Q
B	1190	*orf1ab*	C	C	C	C	T	3		P308S
C	5084	*orf1ab*	A	A	A	A	G	2		A1606T
D	9438	*orf1ab*	C	C	C	C	T	3		T3058I
E	11,083	*orf1ab*	G	T	G	G	T	9		L3606F
F	18,488	*orf1ab*	T	T	T	T	C	2		I6074V
G	21,707	*S*	C	C	N/A	C	T	5		H48Y
H	22,661	*S*	G	G	G	G	T	5		V366F
I	26,144	*orf3*	G	G	G	G	T	18		G251V
J	27,147	*M*	G	G	G	G	C	2		I208T
K	28,077	*orf8*	G	G	G	G	C	4		V61L
L	28,144	*orf8*	C	C	C	T	C	99	38	L84S
M	28,854	*N*	C	C	C	C	T	5		S194L
N	28,878	*N*	G	G	G	G	A	6		S202N
O	29,019	*N*	A	A	A	A	T	2		D249H
P	29,303	*N*	C	C	C	C	T	2		K343I
Synonymous										
α	2662	*orf1ab*	C	T	T	C	T	3		C2397T
β	8782	*orf1ab*	T	T	T	C	T	100	37	C8517T
γ	10,138	*orf1ab*	T	T	T	C	T	134	3	C9873T
δ	15,324	*orf1ab*	C	C	C	C	T	2		C15059T
ϵ	17,373	*orf1ab*	T	C	T	C	T	132	5	C17108T
ζ	18,060	*orf1ab*	T	T	A	C	T	131	6	C17795T
η	18,603	*orf1ab*	T	T	C	T	A	2		T18338C
θ	23,569	*S*	A	C	A	T	C	2		T2007C
ι	23,605	*S*	N/A	N/A	N/A	T	G	2		T2043G
κ	24,034	*S*	T	C	C	C	T	131	6	C2472T
λ	24,325	*S*	A	A	A	A	G	2		A2763G
μ	26,729	*M*	T	T	T	T	C	4		T207C
ν	29,095	*N*	T	T	T	C	T	125	12	C822T

I: Number of changes was inferred by outgroup comparison only

II: Number of changes was cross-referenced with the haplotype network of SARS-CoV-2;only numbers different from method I were shown

*E: envelope; M: matrix; N: nucleocapsid; S: spike*

**Fig. 2:**
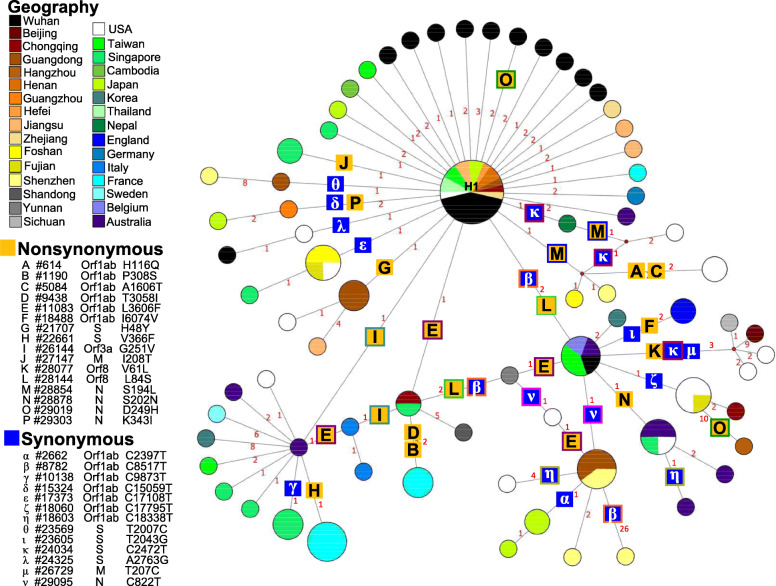
A haplotype network of sampled SARS-CoV-2 genomes. The haplotype network was constructed by the median joining algorithm. Circle areas are proportional to the number of sequences. Numbers along the branches are mutation steps between haplotypes. Mutation types are given on the branches. Mutations involved in different evolutionary pathways or occurred more than once are enclosed. Also see Table 2 for comparison. Seven genomes—EPI_ISL_404253, 407,079, 408,511, 408,512, 408,487, 410,480, and 408,483—were excluded from this analysis because their sequences contained too many ‘N’ notations

To get around the potential problem caused by multiple substitutions, we cross-referenced the course of changes using the SARS-CoV-2 haplotype network (Fig. 2) and phylogeny (Supplementary Fig. 2; Supplementary Table 2). The two analyses yield very different pictures. For example, the highest frequency derived mutation in Table 2 is a C-T synonymous change at 10138 (marked γ in Fig. 2 and Table 2). All three sequences from Singapore share the T nucleotide also found in the RaTG13 outgroup. Using the outgroup comparison, the C found in the rest of the human SARS-CoV-2 sequences is a derived mutation. However, the T at this position is restricted to genomes collected from Singapore on 2/4 and 2/6/2020 and not found in earlier samples. It is thus more sensible to infer that this T is a back mutation derived from C rather than an ancestral nucleotide. Another synonymous change at position 24,034 occurred twice (C24034T) on different genomic backgrounds (marked κ in Fig. 2). Although the outgroup sequence at this position is T, it is more likely that the C at this position is the ancestral nucleotide. We observed a number of such back or repeated mutations. An A-T nonsynonymous change at 29019 (D249H in nucleocapsid protein, marked O in Fig. 2) also occurred twice.

Repeated mutations may be caused by intergenomic recombination. Indeed, the result of four haplotype test suggested that at least two recombination events may have occurred between positions 8782 and 11,083 and between 11,083 and 28,854. We noticed that a sequence isolated on 1/21/2020 from a patient in the United States (EPI_ISL_404253) exhibited Y (C or T) at both positions 8782 and 28,144. Although, the possibility that two novel mutations might have occurred within this patient cannot be 100% ruled out, the alternative explanation that this patient may have been co-infected by two viral strains seems more plausible. After cross-referencing with the haplotype network and the phylogeny, all mutations listed as high frequency in Table 2 and Fig. 1a were re-assigned to the other side of the frequency spectra. We only see an excess of singleton mutations, consistent with a recent origin of SARS-CoV-2 (Fig. 1b) and suggesting that the virus has mainly evolved under constraint.

Perhaps the most controversial case is the T-C change at position 28,814 which alters Leucine (L) to Serine (S) in orf8 protein (L84S). Since both pangolin and RaTG13 have a C at this position (Table 2), Tang et al. suggested that 84 L is derived from 84S in the human virus [13]. The 84S was not discovered until 1/5/2020, by which time 23 SARS-CoV-2 genomes have been sampled. After the first appearance, its frequency gradually increased, reaching approximately 30% by 1/23/2020, suggesting that 84S may exhibit some advantage over 84 L. If genomes carrying 84S were ancestral, it would be a challenge to explain its absence in early samplings. In addition, as mentioned above, C-T transitions are dominant in coronavirus evolution and multiple hits were observed in SARS-CoV-2 (Fig. 2). It is therefore possible that 28814C mutated to T after ancestral SARS-CoV-2 diverged from the common ancestor with RaTG13 and recently changed back to C. Finally, if 84 L is indeed a derived haplotype and has rapidly increased in its frequency by positive selection, we would expect haplotypes carrying 84 L to have accumulated more derived mutations than haplotypes with 84S. However, after correcting for mutational direction, the two haplotypes exhibited similar mutation frequency spectra (Supplementary Fig. 3). The alternative hypothesis that 84S is a back mutation from 84 L is more plausible.

### Selection pressure on SARS-CoV-2

**Fig. 3:**
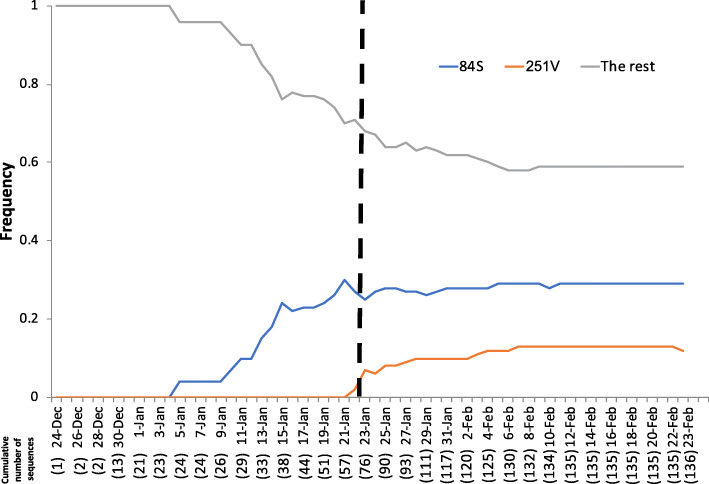
Mutation frequency of 84S in orf8 and 251 V in orf3 proteins. Numbers in parentheses are cumulative number of sequences on the indicated day. The dashed line indicates the date of the Wuhan lockdown

In addition to L84S, a G-T transversion at 26144 which caused an amino acid change in orf3 protein (G251V) is also at intermediate frequency (Table 2). 251 V was first seen on 1/22/2020 and gradually increased its frequency to 13% by our sampling date (Fig. 3). We note that the emergence of 84S in orf8 and 251 V in orf3 proteins are consistent with the lockdown of Wuhan on 1/23/2020. The former first appeared in early January, gradually increased its frequency, and reached a plateau around 1/23/2020. The latter showed up on 1/22/2020 and rapidly increased its frequency within 2 weeks.

Based on Fig. 3, we divided the sampling course into two epidemic episodes, from the first sampled sequence (12/24/2019) to before the lockdown of Wuhan (1/21/2020) and from 1/22/2020 to the date of the last sequence sampling (2/23/2020). The dN/dS of coding regions within the two episodes were estimated. As roughly 87% of mutations were singletons, many of these are probably sequencing errors, affecting synonymous and nonsynonymous sites equally and inflating our dN/dS estimates. In addition, since dN/dS is already extremely small in SARS-CoV-2 (Table 1), such inflation would have a large effect on dN/dS estimates. We therefore estimated dN and dS with (Supplementary Table 3) and without singletons (Table 3).

**Table 3:** Comparison of dN, dS, and dN/dS estimates in the coding regions of SARS-CoV-2 without singleton between two episodes

Gene	Episode I (*N* = 57) (2019/12/24–2020/1/21)		Episode II (*N* = 79) (2020/1/22–2020/2/23)		Episode I + II (2019/12/24–2020/2/23)	
	dN × 10^4^	dS × 10^4^	dN × 10^4^	dS × 10^4^	dN X 10^4^	dS X 10^4^
	dN/dS		dN/dS		dN/dS	
All	0.34	1.70	0.78	1.98	0.61	1.87
	0.20		0.39		0.32	
*orf1a*	0.10	1.46	0.37	2.15	0.26	1.85
	0.07		0.17		0.14	
*orf1b*	0.06	0.81	0.08	1.60	0.07	1.29
	0.07		0.05		0.05	
*spike*	0.23	2.49	0.64	1.82	0.48	2.10
	0.09		0.35		0.23	
*orf3*	0.00	0.00	**5.30**	(1.98)*	**3.42**	(1.87)*
	0.00		**2.68**		**1.83**	
*envelope*	0.00	0.00	0.00	0.00	0.00	0.00
	0.00		0.00		0.00	
*matrix*	0.00	4.60	0.97	3.37	0.57	3.86
	0.00		0.29		0.15	
*orf6*	0.00	0.00	0.00	0.00	0.00	0.00
	0.00		0.00		0.00	
*orf7*	0.00	0.00	0.00	0.00	0.00	0.00
	0.00		0.00		0.00	
*orf8*	**16.26**	(1.70)*	**15.84**	(1.98)*	**15.90**	(1.87)*
	**9.57**		**8.02**		**8.51**	
*nucleocapsid*	1.16	7.31	2.98	4.26	2.25	5.61
	0.16		0.70		0.40	

*No synonymous mutation in this region was observed. The genome-wide dS value was used here. As the sequence EPI_ISL_411929 from South Korea did not have sampling date, it was excluded from this analysis

The dN/dS of *orf8* gene in episode I and II and *orf3* gene in episode II show strong signatures of positive selection, consistent with increase of 84S and 251 V frequency during these periods, and may suggest a role of adaptation (Table 3). The overall dN/dS within each episode was 5–10 times higher than dN/dS between coronavirus genomes derived from different species (Table 1). The elevated dN/dS of SARS-CoV-2 is either due to its adaptation to human hosts or relaxation of selection. For a recently emerged virus, it is reasonable to expect operation of positive selection at the early stage. In that case, the dN/dS during episode I should be greater than during episode II [33, 34].

When singletons were included, dN/dS in episode I was approximately 20% higher than that in episode II across the genome (Supplementary Table 3). In contrast, we observed the opposite result after removing singletons—i.e., dN/dS in episode I was approximately 50% lower than that in episode II (Table 3). Therefore, the elevation of dN/dS was most probably due to a relaxation in selection. We further divided episode I into Ia and Ib, according to the appearance of 84S in orf8 protein on 1/6/2020. The genome-wide dN/dS values were 0.27 and 0.23 for episode 1a and 1b, respectively (Supplementary Table 4). Therefore, as shown in the frequency spectra, the signature of positive selection is weak at the early stage of the epidemic.

### The origin of SARS-CoV-2

**Fig. 4:**
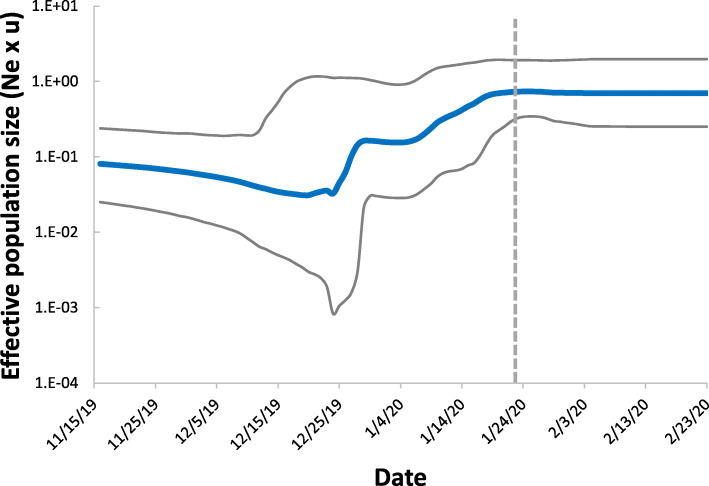
The epidemic growth curve for the SARS-CoV-2 outbreak. The three lines are the median (blue line) and 95% HPD intervals (gray lines) of the Bayesian skyline plot (m = 5). Vertical dash line indicates the date of the Wuhan lockdown

The estimated mutation rate of SARS-CoV-2 is 2.4 × 10^− 3^/site/year with 95% highest posterior density (HPD) of 1.5–3.3 × 10^− 3^/site/year. The mutation rate at the third codon position is 2.9 × 10^− 3^/site/year (95% HPD 1.8–4.0 × 10^− 3^/site/year), which is in a good agreement with synonymous mutation rate of SARS-CoV, 1.67–4.67 × 10^− 3^ /site/year [34]. SARS-CoV-2 is estimated to have originated on 12/11/2019 (95% HPD 11/13/2019–12/23/2019). The initial effective population size of the virus was small, which is consistent with the recent origin of SARS-CoV-2. The population size showed approximately 20-fold increase from the onset of the outbreak to the lockdown of Wuhan (1/23/2020) and ceased to increase afterwards, demonstrating the effectiveness of social distancing on preventing virus spread (Fig. 4). We have to point out that the TMRCA and epidemic growth curve estimation are strongly influenced by the sampling scheme. For example, since the earliest available genome was sampled on 12/24/2019 almost 1 month after the outbreak, the real origin of the current outbreak may actually be earlier than our estimation. In addition, all sequences from Wuhan were sampled before 01/05/2020 which would have an influence on demographic estimation.

We estimated genetic variation, including the number of segregating sites, Watterson’s estimator of θ, and nucleotide diversity (π) of the SARS-CoV-2. Since both π and θ are estimators of 4Nu (N and u are the effective population size and mutation rate, respectively), they should be close to each other at the mutation-drift equilibrium [35]. Because θ is strongly influenced by rare mutations which are common during recent population expansion [24], it is a better estimator of genetic diversity for SARS-CoV-2. For example, when all samples are considered, θ (13.92 × 10^− 4^) is approximately eight times higher than π (1.81 × 10^− 4^, Table 4). Among samples collected from different locations, sequences from China exhibited higher genetic variation in terms of the number of segregating sites, θ and π, than the rest of the world combined, consistent with the observation that the outbreak originated in China, as the source populations are expected to exhibit higher genetic variation than derived populations [35].

**Table 4:** Estimated nucleotide diversity of SARS-CoV-2 across geographic regions

Sample origin	Sample size	S	θ x 10^−4^	π x 10^−4^
Total	137	223	13.92	1.81
China	64	157	11.38	2.10
Wuhan	24	41	3.76	1.16
Rest of China	40	119	9.59	2.62
Rest of the World	73	81	5.71	1.52
USA	17	28	2.84	1.71
Rest of the World excluding USA	56	62	4.63	1.43

S: Number of segregating sites

θ: Nucleotide diversity based on Watterson [29]

π: Nucleotide diversity based on Nei and Li [30]

The haplotype network also supports this notion (Fig. 2). Usually, ancestral haplotypes have a greater probability of being in the interior, have more mutational connections, and are geographically more widely distributed. The H1 haplotype is at the center of the network and is found in four countries and many places in China. In addition, a large portion of haplotypes is directly connected to H1. Therefore, it is likely that H1 is the ancestral haplotype. As 45% of H1 are found in Wuhan, this location is the most plausible origin of the ongoing pandemic.

## Discussion

A close relationship between SARS-CoV-2 and pangolin_2019 at the amino acid level in the RBD region of the spike protein might be due to recent recombination [25, 26], data contamination, or convergent evolution. Since recent recombination and DNA contamination should affect synonymous and nonsynonymous sites equally, they can be convincingly rejected as great divergence at synonymous sites was observed in spite of similar amino acid sequences between the two genomes. While genotypic convergence may be observed in viruses repeatedly evolving under particular conditions, such as drug resistance and immune escape [36–39], it is otherwise rare. For adaptations that do not involve highly specialized conditions, divergent molecular pathways may develop and genotypic convergence would not be expected [40]. For example, SARS-CoV and SARS-CoV-2 both use the spike protein to bind human ACE2 [2], but five out of six critical amino acids within the RBD are different between these two viruses [27]. Since the SARS-CoV-2 and pangolin_2019 have diverged at about 47% of synonymous sites and infect different hosts, the idea that they share five out of six critical amino acids within RBD through convergent evolution seems far-fetched.

We therefore hypothesize that, instead of convergent evolution, the similarity of RBD between SARS-CoV-2 and pangolin_2019 was caused by an ancient inter-genomic recombination. Assuming a synonymous substitution rate of 2.9 × 10^− 3^/site/year, the recombination was estimated to have occurred approximately 40 years ago (95% HPD: 31–69 years; divergence time (t) = divergence (dS)/(substitution rate × 2 × 3), considering dS in RBD is 3-fold of genome average). The amino acids in the RBD region of the two genomes have been maintained by natural selection ever since, while synonymous substitutions have been accumulated. If this is true, SARS-CoV-2 may have circulated cryptically among humans for years before being recently noticed.

The ancient origin of SARS-CoV-2 is supported by its lack of a signature of adaptive evolution as shown by frequency spectra and dN/dS in samples from the recent epidemic. For a recently acquired virus, rapid evolution and a strong signature of positive selection are expected. For example, during its short epidemic in 2002–2003, several rounds of adaptive changes have been documented in SARS-CoV genomes [33, 34]. After adapting to its host, the virus may evolve under purifying or relaxed selection, exactly as we see in SARS-CoV-2. Therefore, it is important to sequence samples from the early outbreak and to examine hospital archives for the trace of SARS-CoV-2 ancestors. This information not only can help us to understand the evolutionary path of this virus but also unravel the critical steps for it to achieve effective spreading in humans.

In addition to the RBD, the SARS-CoV-2 spike protein also contains a small insertion of a polybasic cleavage site which was thought to be unique within the B lineage of betacoronaviruses [27]. However, a recent analysis of bats collected from Yunnan, China, identified a similar insertion in a sequence, RmYN02, closely related to SARS-CoV-2, providing strong evidence that such seemingly sorcerous site insertions can occur in nature [11]. Both the polybasic cleavage site in RmYN02 and RBD in pangolin_2019 suggest that, like with SARS-CoV [6], all genetic elements required to form SARS-CoV-2 may have existed in the environment. More importantly, they can be brought together by frequent intergenomic recombination (see Result). Nature never runs out of material to create new pathogens. It is not whether but when and where the next epidemic will occur.

There is a heated debate about the evolutionary forces influencing the trajectory of the L84S mutation in orf8 protein (http://virological.org/t/response-to-on-the-origin-and-continuing-evolution-of-sars-cov-2/418). While Tang et al. considered Serine is the ancestral amino acid [13], we present evidence that it is a back mutation. The majority of sequences in Wuhan were sampled before early January 2020 and most genomes carrying 84S were found outside Wuhan after middle to late January 2020. The discrepancy in time and space impedes the effort to resolve the debate. It would require more sequences from the early stage of the epidemic to settle this issue. Regardless of its ancestral or derived status, we hypothesize that 84S may confer some selective advantage. Unless the sampling scheme is deliberately skewed, it is difficult to explain such dramatic frequency gain of 84S, from 0 to ~ 30% in 2 weeks. Oddly, its frequency ceased to increase after 1/23/2020, when Wuhan was locked down. This coincidence prompts us to consider the effect of social distancing on virus transmission. Another line of evidence comes from the frequency increase of 215 V in orf3 protein. The 215 V first appeared on 1/22/2020 and rapidly increased its frequency within 2 weeks.

Several studies suggested that the orf8 protein may function in viral replication, modulating endoplasmic reticulum stress, inducing apoptosis, and inhibiting interferon responses in host cells (41–45 [41–45]. During the SARS spread, frequency of several orf8 mutations fluctuated in accordance with different phases of the outbreak, suggesting that *orf8* underwent adaptation during the SARS epidemic [34]. It is suggested that 84S may induce structural disorder in the C-terminus of the protein and may generate a novel phosphorylation target for Serine/Threonine kinases of the mammalian hosts [46].

SARS-CoV orf3 protein has been shown to activate NF-κB and the NLRP3 inflammasome and causes necrotic cell death, lysosomal damage, and caspase-1 activation. In addition, *orf3* is required for maximal SARS-CoV replication and virulence. All of the above likely contributes to the clinical manifestations of SARS-CoV infection [47–49]. Therefore, these two mutations may have some functional consequences and be worth investigating further. By the time we prepared this manuscript, the 215 V frequency ceased to increase. However, a parallel mutation has occurred in a different genomic background, further supporting the idea that this mutation may require further study.

## Conclusion

In contrast to adaptive evolution previously reported for SARS-CoV in its brief epidemic, our analysis of SARS-CoV-2 genomes shows signs of relaxation of selection which, in combination with an ancient intergenomic introgression in RBD of spike protein, suggests that SARS-CoV-2 might have cryptically circulated within humans for years before being recently noticed. Data from the early outbreak and hospital archives are needed to trace its evolutionary path and reveal critical steps required for effective spreading. We found that the lockdown of Wuhan is strongly associated with frequency fluctuations of 84S in orf8 and 215 V in orf3 proteins and population size of the virus, suggesting the effectiveness of human intervention, such as social distancing, on preventing virus spread.

## Supplementary information

**Additional file 1: Supplementary Figure 1.** The neighbor-joining tree of SARS-CoV-2 related coronaviruses constructed by concatenating coding sequences based on the Kimura 2-parameter model implemented in MEGA-X. **Supplementary Figure 2.** Unrooted neighbor-joining tree of SARS-CoV-2 constructed by concatenating coding sequences based on the Kimura 2-parameter model implemented in MEGA-X. Non-singleton changes are shown along the branches. The location of each sequence is given (above the slash) followed by its sampling date (below the slash). For multiple sequences sampled on the same date from the same location, the index, a, b, c, d, and etc. is given. Details are listed in Supplementary Table 2. **Supplementary Figure 3.** Frequency spectra of SARS-CoV-2 carrying 84 L (*n* = 98) (A) and 84S (*n* = 39) (B) in orf8 protein. The direction of changes was cross-referenced with the haplotype network shown in Fig. 2

**Additional file 2: Supplementary Table 1.** People contributed to sequence generation and sharing

**Additional file 3: Supplementary Table 2.**

**Additional file 4: Supplementary Table 3.** Comparison of dN, dS, and dN/dS in the coding regions of SARS-CoV-2 with singleton between different episodes. **Supplementary Table 4.** Comparison of dN, dS, and dN/dS in the coding regions of SARS-CoV-2 without singleton between episode Ia and Ib.

## Data Availability

All genome sequences were downloaded from Global Initiative on Sharing Avian Influenza Data (GISAID, https://www.gisaid.org/) and GenBank (https://www.ncbi.nlm.nih.gov/nucleotide/).
