# Supplementary material for: The origin and underlying driving forces of the SARS-CoV-2 outbreak
Source: J Biomed Sci. 2020 Jun 7;27:73. doi: 10.1186/s12929-020-00665-8 (PMC7276232; doi:10.1186/s12929-020-00665-8)
Supplement: Supplementary file 1 — Additional file 1: Supplementary Figure 1. The neighbor-joining tree of SARS-CoV-2 related coronaviruses constructed by concatenating coding sequences based on the Kimura 2-parameter model implemented in MEGA-X. Supplementary Figure 2. Unrooted neighbor-joining tree of SARS-CoV-2 constructed by concatenating coding sequences based on the Kimura 2-parameter model implemented in MEGA-X. Non-singleton changes are shown along the branches. The location of each sequence is given (above the slash) followed by its sampling date (below the slash). For multiple sequences sampled on the same date from the same location, the index, a, b, c, d, and etc. is given. Details are listed in Supplementary Table 2. Supplementary Figure 3. Frequency spectra of SARS-CoV-2 carrying 84 L (n = 98) (A) and 84S (n = 39) (B) in orf8 protein. The direction of changes was cross-referenced with the haplotype network shown in Fig. 2 [file 12929_2020_665_MOESM1_ESM.docx]

**Supplementary Figures.**

**Supplementary Figure 1.** The neighbor-joining tree of SARS-CoV-2 related coronaviruses constructed by concatenating coding sequences based on the Kimura 2-parameter model implemented in MEGA-X.

**Supplementary Figure 2.** Unrooted neighbor-joining tree of SARS-CoV-2 constructed by concatenating coding sequences based on the Kimura 2-parameter model implemented in MEGA-X. Non-singleton changes are shown along the branches.

The location of each sequence is given (above the slash) followed by its sampling date (below the slash). For multiple sequences sampled on the same date from the same location, the index, a, b, c, d, and etc. is given. Details are listed in Supplementary Table 2.

**Supplementary Figure 3.** Frequency spectra of SARS-CoV-2 carrying 84L (n=98) (A) and 84S (n=39) (B) in orf8. The direction of changes was cross-referenced with the haplotype network shown in Fig. 2
