# Supplementary material for: The origin and underlying driving forces of the SARS-CoV-2 outbreak
Source: J Biomed Sci. 2020 Jun 7;27:73. doi: 10.1186/s12929-020-00665-8 (PMC7276232; doi:10.1186/s12929-020-00665-8)
Supplement: Supplementary file 4 — Additional file 4: Supplementary Table 3. Comparison of dN, dS, and dN/dS in the coding regions of SARS-CoV-2 with singleton between different episodes. Supplementary Table 4. Comparison of dN, dS, and dN/dS in the coding regions of SARS-CoV-2 without singleton between episode Ia and Ib. [file 12929_2020_665_MOESM4_ESM.docx]

**Supplementary Table.**

Table 3. Comparison of dN, dS, and dN/dS in the coding regions of SARS-CoV-2 with singleton between different episodes

| Gene | Episode I (N=57)  (2019/12/24-2020/1/21) | | Episode II (N=79)  (2020/1/22-2020/2/23) | | Episode I+II  (2019/12/24-2020/2/23) | |
| --- | --- | --- | --- | --- | --- | --- |
|  | dN X 10^4^ | dS X 10^4^ | dN X 10^4^ | dS X 10^4^ | dN X 10^4^ | dS X 10^4^ |
|  | dN/dS | | dN/dS | | dN/dS | |
| All | 1.83 | 3.80 | 1.15 | 2.84 | 1.44 | 3.26 |
|  | 0.48 | | 0.40 | | 0.44 | |
| *orf1a* | 1.99 | 3.75 | 0.77 | 2.96 | 1.28 | 3.29 |
|  | 0.53 | | 0.26 | | 0.39 | |
| *orf1b* | 0.55 | 1.97 | 0.36 | 2.09 | 0.44 | 2.05 |
|  | 0.28 | | 0.17 | | 0.21 | |
| *spike* | 2.29 | 4.58 | 0.98 | 2.81 | 1.53 | 3.55 |
|  | 0.50 | | 0.35 | | 0.43 | |
| *orf3* | 3.67 | 2.08 | 7.27 | 2.96 | 6.10 | 2.59 |
|  | **1.76** | | **2.45** | | **2.36** | |
| *envelope* | 0.00 | 0.00 | 0.00 | 0.00 | 0.00 | 0.00 |
|  | 0.00 | | 0.00 | | 0.00 | |
| *matrix* | 1.51 | 9.34 | 0.97 | 3.37 | 1.20 | 5.85 |
|  | 0.16 | | 0.29 | | 0.21 | |
| *orf6* | 0.00 | 0.00 | 0.00 | 0.00 | 0.00 | 0.00 |
|  | 0.00 | | 0.00 | | 0.00 | |
| *orf7* | 3.96 | 4.81 | 0.00 | 0.00 | 1.68 | 1.92 |
|  | 0.82 | | 0.00 | | 0.88 | |
| *orf8* | 17.47 | 5.21 | 16.72 | **(2.84)*** | 16.92 | 2.16 |
|  | **3.35** | | **5.88** | | **7.83** | |
| *nucleocapsid* | 1.51 | 11.44 | 2.98 | 7.85 | 2.40 | 9.43 |
|  | 0.13 | | 0.38 | | 0.25 | |
| As the sequence EPI_ISL_411929 from South Korea did not have sampling date, it was excluded from this analysis. | | | | | | |

Supplementary Table 4. Comparison of dN, dS, and dN/dS in the coding regions of SARS-CoV-2 without singleton between episode Ia and Ib

| Gene | Episode Ia (N=23)  (2019/12/24-2020/1/5) | | Episode Ib (N=34)  (2020/1/6-2020/1/23) | |
| --- | --- | --- | --- | --- |
|  | dN X 10^4^ | dS X 10^4^ | dN X 10^4^ | dS X 10^4^ |
|  | dN/dS | | dN/dS | |
| All | 0.04 | 0.13 | 0.47 | 2.08 |
|  | 0.27 | | 0.23 | |
| *orf1a* | 0.00 | 0.00 | 0.16 | 1.58 |
|  | 0.00 | | 0.10 | |
| *orf1b* | 0.00 | 0.00 | 0.09 | 1.16 |
|  | 0.00 | | 0.08 | |
| *spike* | 0.00 | 0.98 | 0.39 | 3.09 |
|  | 0.00 | | 0.13 | |
| *orf3* | 0.00 | 0.00 | 0.00 | 0.00 |
|  | 0.00 | | 0.00 | |
| *envelope* | 0.00 | 0.00 | 0.00 | 0.00 |
|  | 0.00 | | 0.00 | |
| *matrix* | 0.00 | 0.00 | 0.00 | 0.00 |
|  | 0.00 | | 0.00 | |
| *orf6* | 0.00 | 0.00 | 0.00 | 0.00 |
|  | 0.00 | | 0.00 | |
| *orf7* | 0.00 | 0.00 | 0.00 | 0.00 |
|  | 0.00 | | 0.00 | |
| *orf8* | 0.00 | 0.00 | **21.31** | **(2.08)*** |
|  | 0.00 | | **10.23** | |
| *nucleocapsid* | **0.82** | **0.13** | 1.43 | 10.92 |
|  | **6.35** | | 0.13 | |
| *No synonymous mutation in this region was detected. The genome-wide dS value was used here. | | | | |
